# *α*-Glucosidase Inhibitor Can Effectively Inhibit the Risk of Tuberculosis in Patients with Diabetes: A Nested Case-Control Study

**DOI:** 10.1155/2020/8085106

**Published:** 2020-05-19

**Authors:** Kai-Huang Lin, Ci-Wen Luo, Shih-Pin Chen, Dom-Gene Tu, Ming-Shian Lin, Yu-Hsiang Kuan

**Affiliations:** ^1^Division of Critical Care Medicine, Department of Internal Medicine, Changhua Christian Hospital, Changhua, Taiwan; ^2^Division of Chest Medicine, Department of Internal Medicine, Changhua Christian Hospital, Changhua, Taiwan; ^3^Department of Pharmacology, School of Medicine, Chung Shan Medical University, Taichung, Taiwan; ^4^Department of Pharmacy, Chung Shan Medical University Hospital, Taichung, Taiwan; ^5^Department of Internal Medicine, School of Medicine, Chung Shan Medical University, Taichung, Taiwan; ^6^Department of Internal Medicine, Chung Shan Medical University Hospital, Taichung, Taiwan; ^7^Department of Nuclear Medicine, Ditmanson Medical Foundation Chia-Yi Christian Hospital, Chiayi, Taiwan; ^8^Department of Biomedical Science, National Chung Cheng University, Chiayi, Taiwan; ^9^Department of Internal Medicine, Ditmanson Medical Foundation Chia-Yi Christian Hospital, Chiayi, Taiwan; ^10^Department of Respiratory Care, Chang Gung University of Science and Technology, Chiayi, Taiwan

## Abstract

Diabetes mellitus (DM) and tuberculosis (TB) are major public health and economic burdens. DM increases Mycobacterium tuberculosis (M.tb) infection rates and treatment durations. This study evaluated the relationship between five classes of oral DM medications and TB infection risk in DM patients. We used longitudinal records from the Taiwan Longitudinal Health Insurance Research Database. DM patients were identified using the International Classification of Diseases, Ninth Revision, Clinical Modification (ICD-9-CM) code 250 and A code A181. TB patients were identified using ICD-9-CM code 010.x-017.x. Oral DM medications were divided into five classes: sulfonylureas, biguanides, meglitinides, *α*-glucosidase inhibitors (AGIs), and thiazolidinediones. Users were classified as nonusers, low-concentration users, and high-concentration users. The incidence rate ratio (IRR) was derived using multivariate Poisson regression to calculate the relative risk of TB infection. DM patients using low- and high-concentration AGIs had significantly lower TB infection risks compared with nonusers. The IRRs of the sulfonylureas and AGI users were [CI] 0.693–0.948) and (95% CI 0.651–0.995), respectively. The other four classes of medications exhibited no significant effect on TB infection risk in DM patients. Furthermore, DM patients using high-concentration AGIs had a significantly lower TB infection risk compared with those using low-concentration AGIs (IRR 0.918, 95% CI: 0.854–0.987). We noted a dose-response relationship in the effects of DM medications on TB risk. Accordingly, we suggest that DM patients use AGIs to benefit from their protective effect on TB infection risk.

## 1. Introduction

Diabetes mellitus (DM) associated with immune system dysfunction, including altered cytokine and chemokine levels, results in Mycobacterium tuberculosis (M.tb)—a primary infection—and the reactivation of latent tuberculosis (TB) [1, 2]. Therefore, DM leads to a higher M.tb infection rate and longer treatment duration [1, 2]. In patients with DM, the risk of TB disease is higher than those of human immunodeficiency virus and TB coinfection, and the incidence of TB in patients with DM is three times greater than that in people without DM [3–5]. High-TB-burden countries, including China, India, Brazil, Bangladesh, Indonesia, and Russia, also present the highest prevalence of DM worldwide [3]. The World Health Organization (WHO) reported that DM is a critical, reemerging, and recognised risk factor for TB [3, 5]. The global prevalence has increased by 20% in less than 30 years and estimates that approximately 642 million people will have DM by 2040 [3, 6]. In Taiwan, DM and its related complications constitute a major public health problem and economic burden because of the accompanying treatment and high healthcare costs, including medication and hospitalisation costs [7]. Patients with DM will be at an increased risk of TB in high-TB-burden countries in 2040 [3–5]. The complex interrelationship between DM and TB remains a challenging issue in public health and clinical medicine [8–10].

Oral DM medications can be categorised into five distinct classes, namely, sulfonylureas, biguanides, meglitinides, *α*-glucosidase inhibitors (AGIs), and thiazolidinediones (TZDs). Of these drugs, sulfonylureas, biguanides, and meglitinides are the most extensively used in DM patients with or without TB [11]. The intracellular growth of M.tb can be attenuated by metformin (MET), which is an oral antidiabetic derivative of biguanides [12]. *In vitro* assays have demonstrated that acarbose, an oral antidiabetic agent belonging to the class of *α*-glucosidase inhibitors, can be effective against peptidyl-prolyl isomerase B and *murE* expression in M.tb [13, 14]. In addition, rifampin is a potent cytochrome P450 (CYP3A) inducer and reduces plasma concentrations of sulfonylureas, TZDs, and meglitinides [11, 15]. Therefore, meglitinides and acarbose have potential for use as adjuvant therapy for TB. To date, no evidence exists regarding the relationship between the five classes of oral DM medications and the risk of TB in patients with DM. Accordingly, the purpose of the present study was to explore this relationship through a nested case-control study design using an extensive healthcare database in Taiwan.

## 2. Methods

### 2.1. Data Sources

The Taiwan National Health Insurance Research Database (NHIRD) contains health information on 99% of the population of Taiwan. The NHIRD includes patient demographics, procedures, primary and secondary diagnoses of disease, medical expenditures, and prescriptions. The Taiwan Longitudinal Health Insurance Database (LHID) contains the entire original claim data of 1 million beneficiaries randomly sampled from the NHIRD. The data of the patients in LHID and NHIRD are not statistically significantly different regarding sex distribution (*χ*^2^ = 0.067, df = 1, *P* = 0.796). The study protocol was reviewed and approved by the Institutional Review Board of Chung Shan Medical University Hospital (no. CS215106).

### 2.2. Study Design

**Figure 1:**
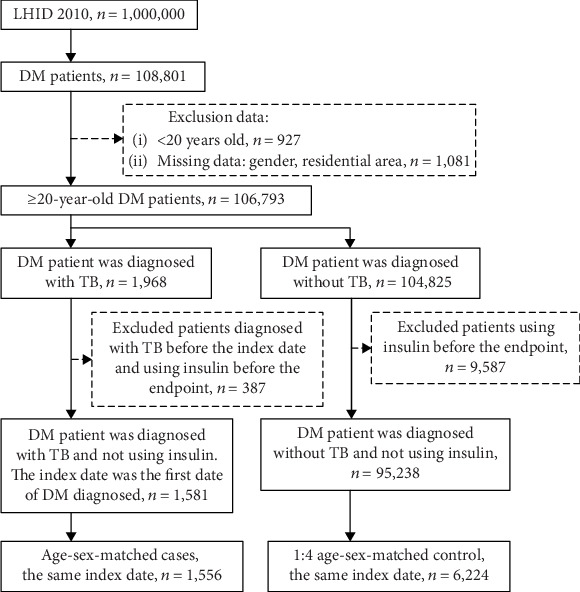
Flow chart of participant selection.

As shown in Figure 1, patients with DM (International Classification of Diseases, Ninth Revision, Clinical Modification (ICD-9-CM) code 250.x; A code, another disease code in Taiwan, has been collected using ICD-9-CM since May 2002 and ICD-9-CM has been fully applied (A181)) who had more than two outpatient diagnoses or more than one discharge diagnosis from 2002 to 2013 were recruited to the study. The index date was the first date that the patients were diagnosed as having DM. Patients with missing data, including sex and residential area, were excluded. The case group comprised patients with DM who were diagnosed as having TB (ICD-9-CM codes 010.x, 011.x, 012.x, 013.x, 014.x, 015.x, 016.x, 017.x, and 018.x) and had more than two outpatient diagnoses or more than one discharge diagnosis in the pulmonology department after the index date, excluding patients who had TB within 6 months. At present, the study enrolled DM patients in past clinical history. The present study was designed as a nested case-control study. Each patient in the case group was matched with 4 patients in the control group by sex, age (±5 y), and index date (±365 d). The case group comprised 1556 patients, and the control group contained 6224 patients. The cumulative dosage of oral diabetes medications was observed between the index date and the endpoint, which were the index date that patients were diagnosed as having TB and the endpoint of the medical record of patients without TB, respectively. Five classes of oral diabetes medication included sulfonylureas, meglitinides, biguanides, AGIs, and TZDs.

### 2.3. Comorbidities

The study endpoint was the diagnosis of TB. Consequently, the relationship between the 5 distinct classes of oral diabetes medications and the risk of TB comorbidities in patients with DM would be analysed. There are pieces of evidence that have purposed that the comorbidities of TB included syphilis (ICD-9-CM codes 090.x, 091.x, 092.x, 093.x, 094.x, 095.x, 096.x, and 097.x); gonococcal infections (ICD-9-CM code 098.x); other venereal diseases (ICD-9-CM 099.x); bacterial, viral, and fungal pneumonias (ICD-9-CM codes 480.x, 481.x, 482.x, 483.x, 484.x, 485.x, and 486.x); empyema (ICD-9-CM code 510.x); emphysema (ICD-9-CM code 492.x); chronic obstructive pulmonary disease (ICD-9-CM codes 490.x, 491.x, 492.x, 493.x, 494.x, 495.x, and 496.x); chronic kidney disease (ICD-9-CM code 585.x); chronic hepatitis (ICD-9-CM code 571.x); intestinal malabsorption (ICD-9-CM code 579.x); ulcerative colitis (ICD-9-CM code 556.x); regional enteritis (ICD-9-CM code 555.x); gastrectomy (based on ICD-9-OP codes and ICD-9-CM codes 43.4.x and 44.31); malignant disease (ICD-9-CM codes 14x, 15x, 16x, 17x, 18x, 19x, 20x, 21x, 22x, and 23x); rheumatoid arthritis (ICD-9-CM code 714.x); disorders involving the immune system (ICD-9-CM code 279.x); and psoriasis (ICD-9-CM codes 696.1, 696.2, and 696.8) [16, 17].

### 2.4. Oral Diabetes Medication Classification

Medications prescribed to patients included in this study were classified into two classes, namely, low- and high-concentration classes, according to their cumulative dosage, with the third quartile value serving as the cutoff point. Medications for which the cumulative dosage was above the third quartile were classified into the high-concentration class, whereas those for which the dosage was below the third quartile were classified into the low-concentration class. Accordingly, the following medications were assigned to the high-concentration class: chlorpropamide > 90000 mg, glibenclamide > 4940 mg, glimepiride > 1080 mg, gliclazide > 59520 mg, tolazamide > 33600 mg, MET > 1076500 mg, buformin > 41100 mg, repaglinide > 510 mg, nateglinide > 30240 mg, acarbose > 32200 mg, miglitol > 4500 mg, rosiglitazone>1500 mg, and pioglitazone > 4200 mg. To further classify oral DM medications in a class, the cumulative dosage of each medication was divided by the third quartile dosage to obtain a ratio. Therefore, for each class, each medication was further classified into a low- or high-concentration class according to the derived ratio, with the cutoff point being the median of the ratios derived in the class. A medication for which the derived ratio was above the median was classified into the high-concentration category, whereas that for which the ratio was below the median was classified into the low-concentration category. Accordingly, the following oral DM medications were assigned to the high-concentration class: sulfonylureas > 2, biguanides > 1, meglitinides > 1, AGIs > 1, and TZDs > 1.

### 2.5. Statistical Analysis

In this nested case-control study, we matched cases to controls according to gender, age, and index date at a 1 : 4 ratio; we used known confounding factors to enhance their comparability. A chi-square test was used to compare the case and control groups in terms of categorical variables, including gender, age, income, and urbanization level, and a two-tailed *t*-test was used to compare the two groups in terms of continuous variables, including age. Incidence density was calculated from the index date to 2013, and the probability of TB occurrence was calculated per month. We used a multivariate Poisson regression model to conduct risk estimation for each patient with DM. Each oral DM medication was used as an interfering factor, and comorbidities, low-income status, length of hospital stay (total number of days spent in the hospital over the entire follow-up period), urbanization level, gender, and age were analysed as covariates. Because the observation time varied for each set of data, we used the time for which patients with DM had TB, a detailed risk estimate, as an offset to determine the relationship between oral DM medication and TB risk. The results are presented as incidence rate ratios (IRRs) and 95% confidence intervals (CIs). Furthermore, we performed a subgroup analysis to explore the differences in TB risk between high- and low-concentration drugs, namely, sulfonylureas, biguanides, meglitinides, AGIs, and TZDs. We conducted another subgroup analysis after adjusting for medications, sex, age, urbanization level, length of hospital stay, low income, and comorbidities. We considered a two-tailed *P* value of <0.05 statistically significant. SAS software was used for data analysis.

## 3. Results

### 3.1. Patient Characteristics

From the Longitudinal Health Insurance Database 2010, we extracted data regarding 95,238 and 1581 cases between 2002 and 2013. We matched cases with controls according to sex, age, and index date at a 1 : 4 ratio. Our sample comprised 6224 controls and 1556 cases between 2002 and 2013. Male patients constituted 67.54% of the sample, and 48.97% of the patients were aged ≥65 years (Table 1). Differences in sex, age group, and mean age between the case (62.71 ± 13.06 years) and control (62.43 ± 12.72 years) groups were not statistically significant. Low-income patients constituted 61.42% and 61.5% of the case and control groups, respectively. Most of the patients in the case (26.22%) and control (29.76%) groups lived in areas with moderate urbanization levels (*P* < 0.05). We observed no statistically significant difference in demographic characteristics, namely, sex, age, and low income, between the case and control groups after matching, except for urbanization level.

As presented in Table 2, the control and case groups were similar with respect to the proportions of patients receiving oral diabetes medication. In the sulfonylurea group, glibenclamide, glimepiride, and gliclazide accounted for the majority of medications (control group, 1973 (31.7%), 2191 (35.2%), and 2185 (35.11%), respectively; case group, 568 (36.5%), 570 (36.63%), and 595 (38.24%), respectively). MET was the main drug used in the biguanide group (control group, 3810 (61.21%); case group, 1015 (65.23%)). Repaglinide was the main drug used in the meglitinide group (control group, 790 (12.69%); case group, 252 (16.2%)). Acarbose was the main medication used in the *α*-glucosidase inhibitor group (control group, 1315 (21.13%); case group, 323 (20.76%)). In the TZD group, rosiglitazone and pioglitazone had similar use distributions (control group, 869 (13.96%) and 833 (13.38%), respectively; case group, 232 (14.91%) and 208 (13.37%), respectively).

### 3.2. Risk of Tuberculosis Infection in Diabetes Medication Users

As presented in Table 3, each variable was included as a predictor in a single model after adjustment for medication, sex, age, urbanization level, length of hospital stay, low income, and comorbidities. No significant difference in the risk of TB infection was observed between patients using low-concentration sulfonylurea and those not using sulfonylurea (IRR 1.154, 95% CI 0.995–1.338). Moreover, no significant difference in the risk of TB infection was observed between users of high-concentration sulfonylurea compared with those nonusers of sulfonylurea (IRR 0.858, 95% CI 0.698–1.055). Among users of high-concentration sulfonylurea, the risk of TB infection exhibited a downward trend, in contrast to the trend observed for nonusers of sulfonylurea. No marked difference in the risk of TB infection was noted between users of low-concentration biguanide and nonusers of biguanide (IRR 1.032, 95% CI 0.887–1.200). However, no significant difference in the risk of TB infection was noted between users of high-concentration biguanide and nonusers of biguanide (IRR 0.904, 95% CI 0.732–1.117). In high-concentration biguanide users, the risk of TB disease had a downward trend in contrast to nonbiguanide users. However, the low- and high-concentration meglitinide users had no significant difference in the risk of TB disease compared to nonmeglitinide users (low concentration: IRR 0.960; 95% CI 0.809–1.138; high concentration: IRR 0.823; 95% CI 0.666–1.016). In the low- and high-concentration meglitinide users, the risk of TB disease had a downward trend in contrast to nonmeglitinide users. A significantly lower risk of TB infection was observed among users of low- and high-concentration AGIs and users of drugs without the *α*-glucosidase inhibitor compared with other patients (low-concentration AGI: IRR 0.810; 95% CI 0.693–0.948; high-concentration AGI: IRR 0.805; 95% CI 0.651–0.995).

Comorbidities associated with a high risk of TB infection included syphilis (IRR 4.599, 95% CI 2.510–8.427) and bacterial, viral, and fungal pneumonias (IRR 2.909, 95% CI 2.531–3.344). Comorbidities associated with a low risk of TB infection included gonococcal infections (IRR 0.184, 95% CI 0.073–0.466) and venereal diseases (IRR 0.428, 95% CI 0.212–0.866).

**Figure 2:**
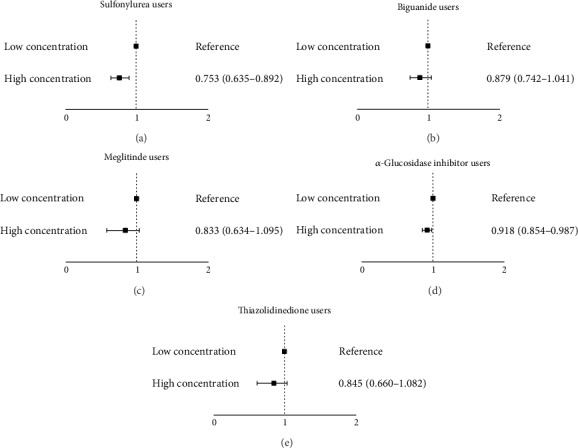
Poisson regression model. (a) Adjusted for meglitinides, biguanides, *α*-glucosidase inhibitor, TZDs, sex, age, urbanization level, length of hospital stay, low income, and comorbidity. (b) Adjusted for sulfonylureas, biguanides, *α*-glucosidase inhibitor, TZDs, sex, age, urbanization level, length of hospital stay, low income, and comorbidity. (c) Adjusted for sulfonylureas, meglitinides, *α*-glucosidase inhibitor, TZDs, sex, age, urbanization level, length of hospital stay, low income, and comorbidity. (d) Adjusted for sulfonylureas, meglitinides, biguanides, TZDs, sex, age, urbanization level, length of hospital stay, low income, and comorbidity. (e) Adjusted for sulfonylureas, meglitinides, biguanides, *α*-glucosidase inhibitor, sex, age, urbanization level, length of hospital stay, low income, and comorbidity.

Figure 2 illustrates the observed differences in the risk of TB infection between high- and low-concentration drugs, adjusted for medication, sex, age, urbanization level, length of hospital stay, low income, and comorbidities. Users of high-concentration drugs had a markedly lower risk of TB infection compared with users of low-concentration drugs that comprised sulfonylurea (IRR 0.753, 95% CI 0.635–0.892; Figure 2(a)) and the *α*-glucosidase inhibitor (IRR 0.918, 95% CI 0.854–0.987; Figure 2(d)). However, the risk of TB infection did not differ significantly between users of high-concentration and users of low-concentration biguanides (IRR 0.879, 95% CI 0.742–1.041; Figure 2(b)), meglitinides (IRR 0.833, 95% CI 0.634–1.095; Figure 2(c)), and TZDs (IRR 0.845, 95% CI 0.66–1.082; Figure 2(e)). In users of high-concentration biguanides, meglitinides, and TZDs, the risk of TB infection exhibited a downward trend compared with that observed for users of low-concentration biguanides, meglitinides, and TZDs.

## 4. Discussion

The WHO reported that in 2018, approximately 10 million new TB cases were recorded, of which 5.7 million involved men and 3.2 million involved women [18]. The number of people with DM increased from 108 million in 1980 to 422 million in 2014 [19]. Evidence reveals that patients with type 2 DM (T2DM) had a higher risk of developing TB in 2019 than they did in 2008 (an increase of 2 to 3 times) [5, 11, 20]. Most medications for T2DM are administered through oral routes [21]. The present study demonstrated that the risk of TB infection could be reduced depending on the class of oral DM medications used. However, the interaction between the different classes of oral DM medications and the resulting decreased risk of TB infection should be addressed in future research. Our nested case-control study primarily revealed that the use of AGIs in patients with DM led to a reduced risk of TB infection.

More than 50% of patients with TB are men [18]. Therefore, the male sex is a major risk factor for TB. Studies conducted on male mice have revealed similar results; that is, male mice were more likely to develop TB [22]. Testosterone and gene polymorphisms on the X chromosome could be susceptibility factors for TB [22, 23]. In the present study, the incidence of TB was higher in male patients. The prevalence of morbidity and mortality associated with TB is higher in people aged older than 65 years in China and the United States [24, 25]. We also observed similar results; that is, older patients with DM had a higher risk of TB infection.

MET, which is structurally associated with biguanides, is the first-line treatment for most patients with T2DM, and sulfonylureas constitute the secondary treatment and are frequently used in the management of T2DM worldwide [26]. MET has been reported to promote M.tb death and to reduce the risk of TB in cell and animal models. Sulfonylureas could reduce the risk of TB in in vitro and animal models [27–30]. MET markedly reduces the risk of TB compared with sulfonylureas in elderly patients with T2DM [31]. Moreover, MET reduces the risk of TB in the first 0.5–3 years of treatment in patients with DM [32–34]. By contrast, our observation performed from 2002 to 2013 revealed that biguanides and sulfonylureas did not significantly reduce the risk of TB infection. However, sulfonylureas significantly reduced the risk of TB in a dose-response relationship. In addition, we discovered that the incidence density and IRRs associated with biguanides were lower than those associated with sulfonylureas. This finding demonstrates that biguanides have superior ameliorative effects to sulfonylureas. These results suggest that MET and sulfonylureas could slightly ameliorate the risk of TB in DM patients aged older than 20 years and followed for more than 10 years.

AGIs, including acarbose and miglitol, specifically and competitively inhibit *α*-glucosidases in the brush border of enterocytes that line the small intestinal villi [35]. AGIs have been used to prevent or delay the onset and development of T2DM syndromes, such as hyperglycaemia and insulin resistance [35]. Acarbose binds strongly to treS, which is involved in basic functions such as energy storage, signalling, and protein protection, and to the bacterial cell wall components of M.tb [36]. Therefore, acarbose is effective as a competitive inhibitor of M.tb treS [36]. *murE* (Rv2158c) is an essential gene of M.tb because it encodes a protein, UDP-*N*-acetylmuramoyl-l-alanyl-d-glutamate:*meso*-diaminopimelate ligase, that catalyses mycobacterial peptidoglycan organisms and synthesises the necessary biochemical reactions. Acarbose strongly binds to murE and inhibits the peptidoglycan biosynthesis of M.tb [36]. Previous clinical trials have not reported the protective effect of AGIs against the risk of TB infection. The present study is the first to indicate that patients with DM who use AGIs have a significantly lower risk of TB infection. Moreover, our results demonstrate a dose-response relationship in the protective effect of AGIs.

Studies have reported that proliferator-activated receptor gamma activation induced by TZDs repressed TB pathogenesis through the activation of macrophages, including CD206 and MCL-1 expression [37, 38]. However, such studies have focused on comparisons of normal cells. TZD-induced ameliorative effects against the risk of TB infection could not be demonstrated in clinical trials. In the present study, we determined that the incidence density and IRRs observed for TZD users were lower than those observed for nonusers. Accordingly, we hypothesised that TZDs have a slight ameliorative effect on the risk of TB infection in DM patients aged older than 20 years and followed for more than 10 years.

Meglitinides, which are insulin secretagogues, are engendered by changes in the permeability of ATP-dependent K^+^ channels in the plasma membrane of *β* cells in pancreatic islets [39]. Meglitinides significantly increase the risk of TB infection in patients with T2DM aged older than 65 years, according to an observation from 1998 to 2009 [40]. By contrast, our results show that meglitinides slightly reduced the risk of TB infection in patients with DM aged older than 20 years, according to our observation from 2002 to 2013. This is probably because the matching of DM patients with those with DM and TB at a 1 : 4 ratio according to gender, age, and index date increased sensitivity in our study. In addition, we adjusted the observed duration as a covariate to reduce time errors regarding the period of medication use. Furthermore, the concentration of meglitinides was divided into three groups, namely, none, low concentration, and high concentration. We expect that this analysis approach approximates and thus reflects clinical situations. These results indicate that TZDs have a slight ameliorative effect against the risk of TB infection in patients with DM.

Studies have explored the relationship between TB and comorbidities. Chronic hepatitis is a notable comorbidity [17]. In the present study, meglitinides had no inhibitory effect on the risk of TB infection in DM patients with chronic hepatitis and TB (not shown in the results). Metabolites from cytochrome P450 result in liver damage [15]. Meglitinides are primarily metabolised by CYP3A4 along with CYP2C8 and organic anion-transporting polypeptides [41, 42]. Meglitinide metabolites may damage the liver and increase the risk of TB infection. Consequently, in the prescription of medications for patients with DM and liver disease, such drugs should be used with caution. Furthermore, we observed that urbanization level and income status had no significant effect on the risk of TB infection. However, elderly DM patients living in rural areas had a higher tendency to develop TB. Ageing is already a risk factor for TB [25]. In rural areas, PM2.5 concentration and lifestyle increase the risk of respiratory diseases, which in turn increases the risk of TB infection [43, 44]. The incidence, morbidity, and mortality rates of TB disease are elevated in low-income populations in several industrialised countries, including Canada, Switzerland, the United States, and Scandinavian countries [45]. The financial burden engendered by the disease is reduced for patients residing in Taiwan owing to the country's National Health Insurance (NHI) system [46]. Therefore, low income is not a major risk factor for TB in Taiwan. The rates of TB-related morbidity and mortality were reported to increase in sub-Saharan African countries [47]. The possible reason is longer hospital stays within 12 months after discharge to a referral hospital. We discovered similar results in the present study; specifically, the risk of TB infection increased with longer hospital stays in patients with DM. DM patients with longer hospitalisation durations may have poorer blood glucose control or more DM complications. Therefore, biologically, they may be more likely to be at risk of TB infection.

The strengths of this study include the categorisation of individual drugs and relative concentrations into five classes of oral DM medications. Classifying the medications into high- and low-concentration classes according to median values could prevent our data from being distorted or skewed by outliers; thus, ratios, intervals, and sequential scales could be determined [48]. Studies have reported similar research methods, that is, adjusting for the use of medications and determining concentrations associated with disease development [49]. Therefore, we believe that our study design can approximate actual clinical settings and yield accurate results.

This study has limitations. No strategies were implemented to determine whether the patients had latent TB infection prior to TB disease diagnosis. Therefore, determining whether TB disease incidence was due to primary progression from direct exposure or to reactivation from a latent M.tb infection was difficult. Latent M.tb exposure was misclassified because of difficulties in the diagnosis of TB disease. Because this study used the National Health Insurance Research Database as a data source, obtaining the relevant characteristics of patients, including their lifestyles, medication use habits, education level, TB severity, and blood glucose concentrations, was difficult. Limited to the International Classification of Diseases, Ninth Revision, Clinical Modification diagnostic codes, directly distinguishing between T1DM, T2DM, and gestational DM for patients aged older than 20 years might have resulted in misclassification bias. To minimise misclassification bias, we mainly used DM for statistical analysis and not for further classification. The exclusion of patients with insulin resistance might have excluded patients with T1DM, but the effect of oral DM medications could be established. Current treatment guidelines are no longer limited to first-line DM medications. Some guidelines recommend supplementing MET with sulfonylurea or gradually adding other types of drugs. For example, compared with regulations in the United Kingdom, Canada, and Australia, Taiwan's NHI regulations are less stringent and do not distinguish between first-line and second-line drugs, which gives doctors the freedom to choose the most suitable drug or combination of drugs for individual patients. Therefore, physicians can choose between first- and second-line medications, which might have caused selection bias. Our study demonstrates that the use of oral DM medications in patients with DM reduces the risk of TB infection. However, we could not derive the survival time of patients with DM; nevertheless, we adjusted for the length of hospital stay as the basis for the patient health index [50]. Through rigorous pairing, we adjusted for cumulative drug concentrations as well as observation time for DM and TB. Taiwan has an intermediate TB burden [51] and high DM burden [52]. Therefore, the findings of the present study should be applicable to countries with similar TB and DM burdens but restricted in countries with lower TB and DM burdens.

The number of people with DM is continually increasing. DM is no longer restricted to elderly people. Young people with DM also require appropriate management strategies and treatment options. Previous research has often compared the use of drugs with TB. Our results demonstrate that in patients with DM, the risk trend for TB decreased after the administration of five oral DM medications. AGIs are the most effective drugs in preventing TB infection. On the basis of our observations, we recommend that clinicians and public health officials prescribe DM drugs in the *α*-glucosidase inhibitor category to patients with pulmonary discomfort in order to reduce the risk of TB infection.

## Figures and Tables

**Table 1:** Demographic characteristics of tuberculosis and nontuberculosis individuals with diabetes.

	Unmatched		*P*	Matched		*P*
	Control	Case		Control	Case	
	*n* = 95238	*n* = 1581		*n* = 6224	*n* = 1556	
Sex						
Female	47649 (50.04%)	516 (32.64%)	<.0001	2020 (32.46%)	505 (32.46%)	1.0000
Male	47579 (49.96%)	1065 (67.36%)		4204 (67.54%)	1051 (67.54%)	
Age						
20-44	51141 (53.7%)	654 (41.37%)	0.0201	604 (9.7%)	151 (9.7%)	1.0000
45-64	17093 (17.95%)	151 (9.55%)		2572 (41.32%)	643 (41.32%)	
≥65	26994 (28.35%)	776 (49.08%)		3048 (48.97%)	762 (48.97%)	
Mean ± SD	56.61 ± 13.42	62.74 ± 13.04		62.43 ± 12.	62.71 ± 13.06	0.4386
Low income						
No	39506 (41.49%)	610 (38.58%)	<0.0001	2435 (38.5%)	610 (38.58%)	0.9539
Yes	55722 (58.51%)	971 (61.42%)		3889 (61.5%)	971 (61.42%)	
Urbanization level						
Highly urbanization	30780 (32.32%)	385 (24.35%)	<0.0001	1835 (29.48%)	376 (24.16%)	<0.0001
Moderate urbanization	29120 (30.58%)	415 (26.25%)		1852 (29.76%)	408 (26.22%)	
Emerging town	14714 (15.45%)	263 (16.64%)		953 (15.31%)	260 (16.71%)	
General town	11902 (12.5%)	243 (15.37%)		906 (14.56%)	241 (15.49%)	
Aged township	1878 (1.97%)	69 (4.36%)		158 (2.54%)	68 (4.37%)	
Agricultural town	3594 (3.77%)	113 (7.15%)		286 (4.6%)	113 (7.26%)	
Remote township	3240 (3.4%)	93 (5.88%)		234 (3.76%)	90 (5.78%)	

Baseline characteristics of study subjects.

**Table 2:** Third quartile of the case and control group (from the index date to the endpoint).

	Control	Case
	*n* = 6224	*n* = 1556
Sulfonylureas		
Chlorpropamide, third quartile = 90000 mg		
None used (0 mg)	6217 (99.89%)	1554 (99.87%)
Low concentration (≤third quartile)	5 (0.08%)	1 (0.06%)
High concentration (>third quartile)	2 (0.03%)	1 (0.06%)
Glibenclamide, third quartile = 4940 mg		
None used (0 mg)	4251 (68.3%)	988 (63.5%)
Low concentration (≤third quartile)	1387 (22.28%)	422 (27.12%)
High concentration (>third quartile)	586 (9.42%)	146 (9.38%)
Glimepiride, third quartile = 1080 mg		
None used (0 mg)	4033 (64.8%)	986 (63.37%)
Low concentration (≤third quartile)	1476 (23.71%)	393 (25.26%)
High concentration (>third quartile)	715 (11.49%)	177 (11.38%)
Gliclazide, third quartile = 59520 mg		
None used (0 mg)	4039 (64.89%)	961 (61.76%)
Low concentration (≤third quartile)	1530 (24.58%)	436 (28.02%)
High concentration (>third quartile)	655 (10.52%)	159 (10.22%)
Tolbutamide, third quartile = 0 mg		
None used (0 mg)	6224 (100%)	1556 (100%)
Low concentration (≤third quartile)	0 (0%)	0 (0%)
High concentration (>third quartile)	0 (0%)	0(0%)
Tolazamide, third quartile = 33600 mg		
None used (0 mg)	6180 (99.29%)	1546 (99.36%)
Low concentration (≤third quartile)	30 (0.48%)	8 (0.51%)
High concentration (>third quartile)	14 (0.22%)	2 (0.13%)
Biguanides		
Metformin, third quartile = 1076500 mg		
None used	2414 (38.79%)	541 (34.77%)
Low concentration (≤third quartile)	2717 (43.65%)	757 (48.65%)
High concentration (>third quartile)	1093 (17.56%)	258 (16.58%)
Buformin, third quartile = 41100 mg		
None used	6211 (99.79%)	1550 (99.61%)
Low concentration (≤third quartile)	9 (0.14%)	4 (0.26%)
High concentration (>third quartile)	4 (0.06%)	2 (0.13%)
Meglitinides		
Repaglinide, third quartile = 510 mg		
None used (0 mg)	5434 (87.31%)	1304 (83.8%)
Low concentration (≤third quartile)	484 (7.78%)	169 (10.86%)
High concentration (>third quartile)	306 (4.92%)	83 (5.33%)
Nateglinide, third quartile = 30240 mg		
None used (0 mg)	6062 (97.4%)	1494 (96.02%)
Low concentration (≤third quartile)	105 (1.69%)	35 (2.25%)
High concentration (>third quartile)	57 (0.92%)	27 (1.74%)
*α*-Glucosidase inhibitor		
Acarbose, third quartile = 32200 mg		
None used (0 mg)	4909 (78.87%)	1233 (79.24%)
Low concentration (≤third quartile)	855 (13.74%)	215 (13.82%)
High concentration (>third quartile)	460 (7.39%)	108 (6.94%)
Miglitol, third quartile = 4500 mg		
None used (0 mg)	6181 (99.31%)	1542 (99.1%)
Low concentration (≤third quartile)	18 (0.29%)	9 (0.58%)
High concentration (>third quartile)	25 (0.4%)	5 (0.32%)
Thiazolidinediones		
Rosiglitazone, third quartile = 1500 mg		
None used (0 mg)	5355 (86.04%)	1324 (85.09%)
Low concentration (≤third quartile)	599 (9.62%)	175 (11.25%)
High concentration (>third quartile)	270 (4.34%)	57 (3.66%)
Pioglitazone, third quartile = 4200 mg		
None used (0 mg)	5391 (86.62%)	1348 (86.63%)
Low concentration (≤third quartile)	504 (8.1%)	129 (8.29%)
High concentration (>third quartile)	329 (5.29%)	79 (5.08%)

Population distribution of drug concentration.

**Table 3:** Adjusted incidence rate ratio of diabetes in patients with and without tuberculosis.

	No. of TB infection events	Observed person-months	Incidence density (95% CI) per 1000 person-months	IRR (95% CI)
Sulfonylureas				
None used	558	153578	3.63 (3.33-3.94)	Reference
Low concentration	716	183274	3.91 (3.61-4.20)	1.154 (0.995-1.338)
High concentration	282	117858	2.39 (2.11-2.68)	0.858 (0.698-1.055)
Biguanides				
None used	540	143596	3.76 (3.44-4.08)	Reference
Low concentration	756	196441	3.85 (3.57-4.13)	1.032 (0.887-1.200)
High concentration	260	114673	2.27 (1.99-2.55)	0.904 (0.732-1.117)
Meglitinide				
None used	1270	370406	3.43 (3.24-3.62)	Reference
Low concentration	179	47573	3.76 (3.20-4.33)	0.960 (0.809-1.138)
High concentration	107	36731	2.91 (2.35-3.48)	0.823 (0.666-1.016)
*α*-Glucosidase inhibitor				
None used	1229	331859	3.70 (3.49-3.91)	Reference
Low concentration	213	73628	2.89 (2.50-3.29)	0.810 (0.693-0.948)
High concentration	114	49223	2.32 (1.88-2.75)	0.805 (0.651-0.995)
Thiazolidinediones				
None used	1206	331516	3.64 (3.43-3.85)	Reference
Low concentration	217	65440	3.32 (2.87-3.77)	0.927 (0.789-1.087)
High concentration	133	57754	2.30 (1.90-2.70)	0.874 (0.707-1.079)
Urbanization level				
Highly urbanization	376	129137	2.91 (2.61-3.21)	1.039 (0.901-1.198)
Moderate urbanization	408	135752	3.01 (2.71-3.30)	Reference
Emerging town	260	68955	3.77 (3.30-4.24)	1.105 (0.943-1.294)
General town	241	65674	3.67 (3.20-4.14)	1.03 (0.872-1.216)
Aged township	68	12801	5.31 (4.02-6.60)	1.215 (0.927-1.593)
Agricultural town	113	23849	4.74 (3.85-5.63)	1.206 (0.968-1.502)
Remote township	90	18542	4.85 (3.83-5.88)	1.04 (0.823-1.316)
Length of hospital stay				
0	1386	423812	3.27 (3.09-3.45)	Reference
1-30	132	26204	5.04 (4.16-5.91)	1.164 (0.967-1.403)
31-60	19	2927	6.49 (3.51-9.47)	1.959 (1.236-3.104)
≥61	19	1767	10.75 (5.82-15.69)	1.636 (1.026-2.606)
Low income				
No	610	34538	3.53 (3.25-3.82)	Reference
Yes	958	282011	3.40 (3.18-3.62)	0.997 (0.891-1.116)
Comorbidity (reference: without)				
Syphilis	42	2029	20.70 (14.31-27.09)	4.599 (2.510-8.427)
Gonococcal infections	7	549	12.75 (3.11-22.39)	0.184 (0.073-0.466)
Venereal diseases	26	1181	22.02 (13.38-30.65)	0.428 (0.212-0.866)
Bacterial, viral, and fungal pneumonias	945	55339	17.08 (15.97-18.19)	2.909 (2.531-3.344)
Empyema	25	1072	23.32 (13.99-32.65)	1.124 (0.748-1.690)
Emphysema	58	3295	17.60 (12.98-22.23)	0.707 (0.537-0.931)
COPD	949	53283	17.81 (16.65-18.97)	3.208 (2.777-3.706)
Chronic kidney disease	382	23878	16.00 (14.36-17.64)	1.375 (1.210-1.562)
Chronic hepatitis	633	36096	17.54 (16.14-18.93)	1.907 (1.696-2.144)
Intestinal malabsorption	7	593	11.80 (2.88-20.73)	1.183 (0.544-2.575)
Ulcerative colitis	10	510	19.61 (7.210-32.01)	1.204 (0.627-2.309)
Regional enteritis	95	5361	17.72 (14.08-21.36)	1.282 (1.034-1.590)
Gastrectomy	5	212	23.58 (2.49-44.68)	2.986 (1.209-7.373)
Malignant disease	592	35611	16.62 (15.26-17.99)	1.646 (1.463-1.852)
Rheumatoid arthritis	137	8079	16.96 (14.06-19.86)	1.475 (1.224-1.777)
Immune mechanism	22	1543	14.26 (8.18-20.34)	1.026 (0.660-1.595)
Psoriasis	61	3542	17.22 (12.81-21.63)	1.317 (1.014-1.709)
Comorbidity (reference: without)				
Syphilis	42	2029	20.70	4.599 (2.510-8.427)
Gonococcal infections	7	549	12.75	0.184 (0.073-0.466)
Venereal diseases	26	1181	22.02	0.428 (0.212-0.866)
Bacterial, viral, and fungal pneumonias	945	55339	17.08	2.909 (2.531-3.344)
Empyema	25	1072	23.32	1.124 (0.748-1.690)
Emphysema	58	3295	17.60	0.707 (0.537-0.931)
COPD	949	53283	17.81	3.208 (2.777-3.706)
Chronic kidney disease	382	23878	16.00	1.375 (1.210-1.562)
Chronic hepatitis	633	36096	17.54	1.907 (1.696-2.144)
Intestinal malabsorption	7	593	11.80	1.183 (0.544-2.575)
Ulcerative colitis	10	510	19.61	1.204 (0.627-2.309)
Regional enteritis	95	5361	17.72	1.282 (1.034-1.590)
Gastrectomy	5	212	23.58	2.986 (1.209-7.373)
Malignant disease	592	35611	16.62	1.646 (1.463-1.852)
Rheumatoid arthritis	137	8079	16.96	1.475 (1.224-1.777)
Immune mechanism	22	1543	14.26	1.026 (0.660-1.595)
Psoriasis	61	3542	17.22	1.317 (1.014-1.709)

Abbreviation: IRR: incidence rate ratio. Multiple Poisson regression model: adjusted for sex, age, urbanization level, length of hospital stay, low income, and comorbidity. Poisson regression model: adjusted for sex, age, urbanization level, length of hospital stay, low income, and comorbidity.

## Data Availability

The data form NHIRD must be used for research purposes only. All applications should be reviewed by peer experts to ensure the rationality of the research. The data used to support the findings of this study are available from the corresponding author upon request.
